# Level and Timing of Implanon Discontinuation and Associated Factors among Women Who Used Implanon in Andabet District, Public Health Facilities, North-West Ethiopia

**DOI:** 10.1155/2021/6647660

**Published:** 2021-08-06

**Authors:** Gizachew Worku Dagnew, Yared Mulu Gelaw, Melash Belachew Asresie, Zelalem Alamrew Anteneh

**Affiliations:** ^1^Department of Reproductive Health and Population Studies, School of Public Health, College of and Health Science, Bahir Dar University, Bahir Dar, Ethiopia; ^2^Department of Health Service Management, School of Public Health, College of and Health Science, Bahir Dar University, Bahir Dar, Ethiopia; ^3^Department of Biostatistics and Epidemiology, School of Public Health, College of and Health Science, Bahir Dar University, Bahir Dar, Ethiopia

## Abstract

**Background:**

Implanon discontinuation is unacceptably high in developing countries, including Ethiopia. Furthermore, there is an observed problem of high unintended pregnancy after method discontinuation that strides to program failure. Therefore, the purpose of this study was to assess the level and determinants of Implanon discontinuation among women who used Implanon in Andabet district, public health facilities, North-West Ethiopia, 2017.

**Methods:**

Facility-based cross-sectional study design was employed among 537 women from Feb. 03 to April 28, 2017. Study participants were selected using a systematic random sampling technique. A face-to-face interview was employed to collect data. Epi-Info version 7 was used for data entry and SPSS version 20 for analysis. Both descriptive and analytical statistical analysis was computed. On multivariable binary logistic regression, a *p* value of less than 0.05 was used to declare statistical significance.

**Results:**

About 37% of Implanon users have discontinued the method before the intended time. About 86% of them discontinued Implanon before two years of insertion. Women who had no live child (AOR = 2.17, 95% CI: 1.25-3.77), women who did not receive preinsertion counseling (AOR = 1.85, 95% CI: 1.15-2.97), women who developed Implanon-related side effect (AOR = 5.17, 95% CI: 3.18-8.40), and women who did not satisfy by the service provided (AOR = 5.40, 95% CI: 3.04-9.57) had higher odds of Implanon discontinuation. On the other hand, women who received appointment follow-up (AOR = 0.23, 95% CI: 0.13-0.41) had lower odds of Implanon discontinuation.

**Conclusions:**

The level of Implanon discontinuation before its intended time was high in the district. Hence, strengthening preinsertion counseling and appointment follow-up as well as improving the clients' level of service satisfaction could increase Implanon's continuation.

## 1. Introduction

Implanon is a second-generation single-rod progestogen-only contraceptive implant with a length of 40 mm and a diameter of 2 mm containing 68 mg of etonogestrel dispersed in a membrane of ethylene-vinyl acetate. It delivers ENG at a dose sufficient to suppress ovulation in every cycle throughout the 3 years of use [1–3]. This single-rod progestogen subdermal (etonogestrel) is developed as a need to reduce some of the problems associated with the six implant systems, Norplant by 2008, and it is prequalified by the World Health Organization (WHO) in 2010 [2, 4]. Implanon has the most effective implants to prevent pregnancy compared to other short-acting contraceptives [2, 5, 6].

Globally, 7% of married or in-union reproductive-age women were using injectable contraceptives [7, 8]. Even though millions of reproductive-age women were using implants, its contribution among all method mix was not more than 1%. And significant numbers of women discontinue the method before its intended period [7]. Across the world, the percentage of Implanon discontinuation ranges from 3% in Burkina Faso to 27% in Yemen in the first year and 23 percent in Liberia to 69 percent in Yemen at the end of three years of insertion [9].

In 2009, the Ethiopian Federal Ministry of Health (FMOH) launched an Implanon scale-up program to improve the availability of long-acting reversible contraceptive (LARC) methods at the community level. The Integrated Family Health Program (IFHP) supports the MOH to train Health Extension Workers (HEWs) as a cadre of frontline health workers on Implanon insertion [10]. The scale-up program was successful in reaching all communities by LAR. Consequently, Implanon use among married or in-union women was increased from 3.4% in 2011 to 8% in 2016 [11]. Despite this improvement, Implanon discontinuation becomes unacceptably high in different parts of the country and becomes a programming challenge. Studies from Ethiopia showed 16% of women discontinued in the first years of Implanon insertion [11], and it reached 46.5% at the end of three years [12].

Discontinuation while still in need (DWSIN) is particularly problematic, and it leaves women at risk of unwanted pregnancy. Around 15 to 20 percent of LARC users faced with an unwanted pregnancy after three months of discontinuation [9]. The incidence of unintended pregnancy secondary to improper switching of another method after discontinuation was high among low-socioeconomic women in developing countries [13, 14]. Although there is a high number of women who come to health institutions and requesting the removal of Implanon prematurely and its contribution to increasing unwanted pregnancy is high, there is little scientific information available from Ethiopia, particularly in the study areas. This study is aimed at assessing the level of premature Implanon discontinuation and associated factors among women who requested Implanon removal in Andabet district, North-West Ethiopia. The findings of this study may support the Implanon provision program by showing important intervention areas.

## 2. Materials and Methods

The facility-based cross-sectional study design was conducted among women who requested Implanon removal from February 03 to April 28, 2017, in Andabet district, North-West Ethiopia. The district is located 717 km from Addis Ababa, the capital city of Ethiopia. According to the 2016 health office report, the total population of the district was 139,462 (70,944 males and 68,518 females). Of that, 33,332 were female reproductive-age groups (15-49 years). The district has 24 Kebeles (small administrative units), 5 health centers, and 24 health posts.

### 2.1. Eligibility Criteria

All women who requested Implanon removal were included in the study.

### 2.2. Sample Size Determination

The sample size was determined by using the Epi-Info version 7 software by considering the following statistical assumption. Educational status and its odds ratio were taken from a previous study [11].

*Assumption*: two-sided significant level: 95%, power (1-beta): 90%, ratio of sample size: 2, percent not read and write with the outcome: 50.8%, percent with secondary education with outcome 34.9% and odds ratio: 0.51, and continuity correction result = 485 which is the largest sample size from the factors. Then, by adding a 12% nonresponse rate, the final sample size (*n*) is

(1) $$\begin{array}{c} n=485*\frac{1}{1-0.12}=544. \end{array}$$

### 2.3. Sampling Method and Procedures

The calculated sample size was allocated proportionally for each health facility based on the average number of three-month client flow. According to the health facility report that provides Implanon removal service (Andabet health center (HC), Jaragedo HC, Aside-Mariam HC, Gono HC, and Generate-Mariam HC), a total of 1276 women removed Implanon three months before the survey. The sampling interval was allocated by dividing women booked to remove Implanon (1276 clients) to the current sample size (544), which was every two clients. A systematic random sampling technique was employed to select the study participants (fig. 1 from supplementary file).

### 2.4. Operational Definition

*Implanon discontinuation*: removal of the method before three years of insertion [15].

*Not implanon discontinuation*: removal of the method at three-year completion of insertion [15].

### 2.5. Data Collection Procedure and Tools

A structured English version questionnaire was adapted from different works of literature that have a related context to the current study [10, 12, 13] and translated to the local language (Amharic). To assure validity and reliability, the tool content (items), clarity of language use, and comprehensibility were extensively reviewed by experts. Moreover, the questionnaire was pretested among 5% of the study participants out of the study area to check tool consistency, sensitivity, and understandability in the local context. The data were collected by face-to-face interview. Five trained data collectors who have a diploma in health sciences (level-IV) and two supervisors who have a BSc degree in nursing participated in data collection.

### 2.6. Data Management and Analysis

Data were entered in Epi-Info version 7 and then exported to the Statistical package for social sciences (SPSS) version 20.0 software for analysis.

Both descriptive and analytical statistical analysis was done. Bivariate analysis was computed to select the candidate variables for multivariable analysis at *p* value ≤0.2. Variables with a *p* value <0.05 in the multivariable binary logistic regression analysis were considered predictor variables for Implanon discontinuation. The adjusted odds ratio (AOR) with its corresponding 95% confidence interval (CI) was used to show the strength of association. Multicollinearity between each explanatory variable was checked by using standard errors. The chi-square assumption was also checked with the contingency tables of the expected and observed frequency. Model fitness was checked by using the Hosmer-Lemeshow goodness of fit test (*p* > 0.05) [15].

## 3. Results

### 3.1. Sociodemographic and Reproductive Characteristics of the Study Participants

A total of 537 women participated in the survey, making a response rate of 98.7%. The age ranges of the participants were between 16 and 47 years with the mean (±SD) age of 29.38 ± 6.30 years. The majority of the respondents were 498 (92.7%) married, 527 (98.1%) Orthodox in religion, and 432 (80.4%) housewives in occupation (Table 1).

### 3.2. Family Planning Service-Related Characteristics of the Study Participants

Before Implanon use, 97.2% of women had information for at least one type of contraceptive method; 519 (96.6%) women had information about injectable, 446 (83.1%) about pills, 117 (21.8%) about condom, 79 (14.7%) about IUCD, and 23 (4.3%) women had information about permanent family planning methods. Most women got information from health professionals. Four hundred forty-four (82.7%) women had ever used other contraceptive methods before using Implanon. 90% of them used injectable contraceptives.

Four hundred seventeen women (77.6%) reported that the long-term duration of protection from unwanted pregnancy was their reason for choosing to use Implanon. Hence, 94 (17.5%) women reported that the unavailability of other method choice was their reason for choosing to use Implanon (Table 2).

### 3.3. Level and Time for Implanon Discontinuation

**Figure 1:**
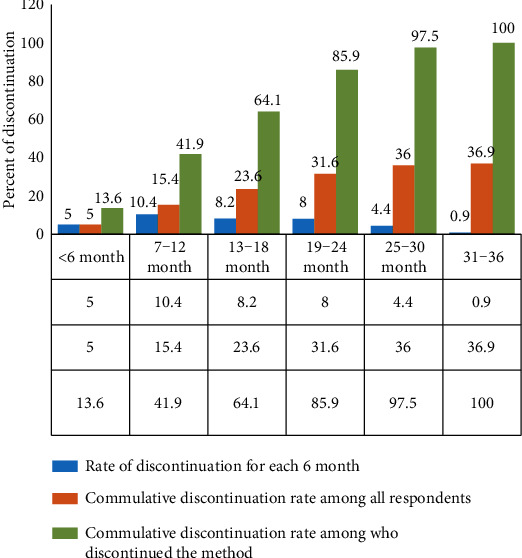
Rate of Implanon discontinuation among women who requested the removal of Implanon in Andabet district, public health facilities; North-West Ethiopia, 2017.

According to this study, out of the total 537 women who requested Implanon removal, 198 (36.9%) (*p* = 36.9%, 95% CI: 33.0%-41.2%) women discontinued the method before its intended period. The most reported reasons for discontinuation were the presence of side effects 136 (68.7%) followed by want to be pregnant 55 (27.7%) (fig. 2 from supplementary-file). The discontinuation of Implanon starts as early as 01 month and as long as 35 months with a mean (±SD) of 15.56 ± 7.82 months of use (Figure 1).

### 3.4. Factors Associated with Implanon Discontinuation

In multivariable binary logistic regression analysis, there were a higher odds of Implanon discontinuation among women who had no live child (AOR = 2.17, 95% CI: 1.25-3.77), had no history of abortion (AOR = 2.62, 95% CI: 1.18-5.44), women felt happy if pregnant soon compared to who felt sad (AOR = 2.66, 95% CI: 1.59-4.45), did not receive preinsertion counseling on potential side effects (AOR = 1.85, 95% CI: 1.15-2.97), developed Implanon-related side effect (AOR = 5.17, 95% CI: 3.18-8.40), and women who were not satisfied by the service given (AOR = 5.40,95% CI: 3.04-9.57) compared to their counterparts. On the other hand, women who received follow-up services have lower odds of Implanon discontinuation (AOR = 0.23, 95% CI: 0.13-0.41) (Table 3).

## 4. Discussions

This study examined the prevalence of Implanon discontinuation ad and associated factors among women who requested Implanon removal in Andabet district, North-West Ethiopia.

The overall discontinuation of Implanon was 36.9% (95% CI: 33.0%-41.2%) with 15.56 ± 7.82 months' mean duration of use. This finding is consistent with the study conducted in Kucha district, Southern Ethiopia (34%) [16], Ghana (40%) [17], and India (37%) [18]. However, this finding is higher than the study done in Zaria (19%) [19], Ilorin in Nigeria (26.1%) [20], and the US population (25.2%) [21]. The first plausible reason for this difference might be due to a difference in preinsertion counseling and follow-up services. During counseling, women can get detailed information about Implanon to correct misconceptions [22, 23]. The second reason might be different in women's experience for Implanon use. All participants in Zaria, Nigeria, had a history of Implanon use [22]. However, in the current study, all participants were new to this method. Women's previous experience with Implanon can help them to tolerate minor side effects for the second use [24]. On the other hand, this finding is lower than the study done in Debretabor Town (65%) [25], Hawassa, Southern Ethiopia (49.3%) [26], and Uganda (56%) [27]. The possible reason for the lower discontinuation rate in this study might be sociodemographic differences of respondents across the study areas. The number of living children might contribute to this lower Implanon discontinuation; in the current study, 82.7% of respondents have living children whereas 65% from the study done at Debretabor Town [25]. This might insist the women discontinue the method before the intended time for giving birth. A desire for pregnancy is one of the main reasons for contraceptive discontinuation [12, 25, 28]. The other possible reason might also be the difference in the accessibility of other alternative contraceptives. In Ethiopia, most urban women have better contraceptive access compared to the rural women [29]. Since the current study participant is rural women, they might have limited access to other alternative contraceptives, and this might insist them to tolerate minor side effects of Implanon as compared to the aforementioned urban-based studies [25, 26].

According to the findings of this study, the predominant reason for Implanon discontinuation reported by the women was Implanon-related side effects followed by the desire for pregnancy. This is similar to other studies in Ethiopia [12, 28]. However, this finding was different from the studies from Jos, Central Nigeria [30]. The possible reason might be different in women's menstrual condition. About 85.5% of women have regular menstruation in Jos, Central Nigeria [30], and only 20.5% of respondents have had regular menstruation in the current study. For many women, menstrual bleeding is the main reason for contraceptive discontinuation [24, 31, 32].

In this study, women who had a history of abortion had low odds of Implanon discontinuation compared to their counterparts. This finding is supported by the study conducted in seven different countries [33]. The possible explanation for this might be due to information and understanding differences about unintended pregnancy, abortion, and related complications. Women who have a history of abortion might learn from their previous bad experiences.

Implanon discontinuation has higher odds among women who had no live children compared to their counterparts. This is similar to the study done in Debre Markos town [12]. In most cases, women who have no live children have more fertility desires compared to those who have live children. Those women might also be influenced by their families to give birth before they become infertile [34–36].

Other findings of this study showed women who reported that they felt happy if they become pregnant soon have high odds of Implanon discontinuation compared to those who felt sad. This is supported by a study done in Ofla district and Jos, Central Nigeria [13, 30]. The desire for childbirth is a common reason for contraceptive discontinuation. This also reflects women's commitment to preventing unintended pregnancy. Those women who committed to preventing unintended pregnancy might tolerate minor side effects of the method [37, 38].

Women who did not receive preinsertion counseling about the potential side effects of the method had high odds of Implanon discontinuation compared to those who received counseling. This finding is supported by the study done in Debre Markos town [12]. The possible reason might be women who are counseled about side effects can develop preset mind about it and be able to tolerate possible minor side effects. Moreover, the information provided during counseling can clear the women's misconceptions that might cause discontinuation [39].

In this study, there are high odds of Implanon discontinuation among women who had not satisfied with the service provided compared to those who were satisfied. This is consistent with studies done in Ethiopia [11, 12, 16]. This might be true that service satisfaction at first contact can increase the continuation of a longitudinal type of service use [40, 41]. Service satisfaction can increase the affection between women and service providers. This enables women to perform what the providers said before discontinuing the method and can tolerate minor side effects of the methods, because those women can develop confidence due to caring professionals that treating possible side effects early [42].

Appointment follow-up had a statistical association with Implanon discontinuation. Women who had an appointment follow-up service have a lower odds of discontinuing the method compared to their counterparts. This is similar to other studies [11, 43]. During follow-up, women may get further detailed counseling and information regarding women's concerns or misconceptions about the method. Furthermore, those women can get management for the developed side effects and may continue with management.

In the current study, the primary reason for discontinuation was facing Implanon-related side effects. The odds of Implanon discontinuation among women who developed side-effect were higher compared to their counterparts. This finding is consistent with the study conducted in Debre Markos and Ofla district [11, 12]. This might be women's intolerance to the newly occurring minor side effects of the method. The other reason might be women who develop vaginal bleeding may interfere with their sexual experience and feel guilt by the usual occurrence of bleeding.

The current study has some limitations; since most variables are assessed retrospectively, there may be recall bias (the women may not remember some information conducted during Implanon insertion). The other limitations are the survey was assessed by using the quantitative data and the lack of women's exploration of the methods and type of services they used.

## 5. Conclusions

The level of Implanon discontinuation in this study was high. Further study is recommended to investigate its contribution to the total unwanted population fertility. The main reasons for discontinuation were method-related side effects followed by a desire for childbirth. Having children at the time of insertion, history of abortion, feeling of women if got pregnant, preinsertion counseling, developed side effects, received appointment follow-up, and perceived service satisfaction were the predictors for Implanon discontinuation. Strengthening contraceptive counseling and appointment follow-up services might increase Implanon continuation. Working to improve clients' perceived satisfaction could also reduce Implanon discontinuation.

## Figures and Tables

**Table 1:** Sociodemographic characteristics of women who requested the removal of Implanon in Andabet district, public health facilities, North-West Ethiopia, 2017.

Variables (*n* = 537)	Frequency	Percentage
*Age* ^∗^		
<20	53	9.9
21-24	66	12.3
25-29	149	27.7
30-34	155	28.9
>35	114	21.2
*Marital status*		
Married	498	92.7
Single	29	5.4
Others (widowed and divorce)	10	1.9
*Educational status*		
Have no formal education	406	75.6
Primary education	61	11.4
Secondary education	26	4.8
Certificate and above	44	8.2
*Husband/partner education (n* = 516)		
Have no formal education	398	77.1
Primary education	51	9.9
Secondary education	18	3.5
Certificate and above	49	9.5
*Religion*		
Orthodox	527	98.1
Muslim	10	1.9
*Occupation*		
Housewife	432	80.4
Merchant	42	7.8
Government employee	37	6.9
Farmer	24	4.5
Student	2	0.4
*Have live children*		
Yes	444	82.7
No	93	17.3
*Need more children (n* = 444)		
Yes	348	78.4
No	96	21.6
*History of abortion*		
Yes	58	10.8
No	479	89.2
*Perceived her spouse wants to give birth in the next two years (n* = 516)		
Yes	261	50.6
No	255	49.4
*Purpose of using contraceptive*		
Spacing	437	81.4
Limiting	100	18.6
*Feeling of women if pregnant soon*		
Being happy	179	33.3
Neutral	148	27.6
Being sad	210	39.1

^∗^The age classification was done based on the previous studies [11, 12, 15].

**Table 2:** Family planning service-related characteristics of women who requested the removal of Implanon in Andabet district, public health facilities, North-West Ethiopia, 2017.

Variables (*n* = 537)	Frequency	Percentage
*Ever heard about contraceptive*		
Yes	522	97.2
No	15	2.8
*Ever used any contraceptive before Implanon use*		
Yes	444	82.7
No	93	17.3
*Preinsertion counseling*		
Yes	393	73.2
No	144	26.8
*Counseling type (n* = 393)		
Individual	327	83.2
Mass counseling	42	10.7
Couple	24	6.1
*Topics discussed during counseling (n* = 393)		
Benefit (advantage)	327	83..2
Side effect	193	49.1
Duration of action	312	79.4
Effectiveness	107	27.2
Time of insertion and removal	270	68.3
*Discuss with husband/partner to use Implanon*		
Yes	349	65.0
No	188	35.0
*Women accompanied by at the time of service provision*		
No one accompanied	417	77.7
Husband/partner	91	16.9
Mother	16	3.0
Others	13	2.4
*Who insist you use Implanon?*		
Women herself	317	59.0
Couple together	79	14.7
Health provider	74	13.8
Husband/partner	67	12.5
*Place of Implanon insertion*		
Health post	290	54.0
Health center	247	46.0
*Reason for using Implanon*		
The long duration of action	417	77.6
Need low follow-up time	307	57.2
Unavailability of other methods	94	17.2
Less side effect	60	11.2
Effectiveness	25	4.7
*Have appointment follow-up after Implanon insertion*		
Yes	151	28.1
No	386	71.9
*Perceived satisfaction by the service provided*		
Yes	435	81.0
No	102	19.0

**Table 3:** Factors associated with discontinuation of Implanon among women who requested the removal of Implanon in Andabet district, public health facilities, North-West Ethiopia, 2017.

Variables (*n* = 537)	Implanon discontinuation		COR (95% CI)	AOR (95% CI)
	Yes	No		
*Educational status*				
No formal education	141	265	1	1
Primary	20	41	0.92 (0.52-1.63)	1.27 (0.58-2.81)
Secondary	14	12	2.19 (0.99-4.87)	2.50 (0.91-6.88)
Certificate and above	23	21	2.06 (1.10-3.85)	1.71 (0.70-4.16)
*Living children*				
Yes	153	288	1	1
No	45	51	1.66 (1.06-2.60)	2.17 (1.25-3.77)^∗^
*History of abortion*				
Yes	14	44	1	1
No	184	295	1.96 (1.05-3.68)	2.62 (1.18-5.44)^∗^
*Feeling of women if pregnant soon*				
Happy	88	91	2.48 (1.63-3.78)	2.66 (1.59-4.45)^∗∗^
Neutral	51	97	1.35 (0.86-2.12)	1.09 (0.63-1.91)
Will be sad	59	151	1	1
*Purpose of using the method*				
For spacing	172	265	1.85 (1.14-3.00)	1.21 (0.59-2.49)
For limiting	26	74	1	1
*Main decider to use Implanon*				
Women her self	108	209	1	1
Husband/partner	27	40	1.31 (0.76-1.31)	0.88 (0.45-1.75)
Couple together	20	59	0.66 (0.38-1.15)	0.90 (0.45-1.80)
Service provider	43	31	2.68 (1.60-4.50)	1.71 (0.90-3.24)
*Counseled about benefit*				
Yes	109	206	1	
No	89	123	1.43 (1.00-2.05)	0.96 (0.59-1.57)
*Counseled about side effect*				
Yes	56	135	1	1
No	142	204	1.68 (1.15-2.45)	1.85 (1.15-2.97)^∗^
*Discussed with their partner*				
Yes	120	229	1	
No	78	110	1.35 (0.94-1.94)	1.16 (0.70-1.92)
*Presence of side effect*				
Yes	162	150	5.67 (3.73-8.63)	5.17 (3.18-8.40)^∗∗^
No	36	189	1	1
*Had appointment follow-up*				
Yes	20	131	0.18 (0.11-0.0.30)	0.23 (0.13-0.41)^∗∗^
No	178	208	1	1
*Satisfied by the service provided*				
Yes	119	316	1	1
No	79	23	9.12 (5.48-15.19)	5.40 (3.04-9.57)^∗∗^

Note: ^∗^statistically significant at *p* < 0.05 and ^∗∗^statistically significant at *p* < 0.001.

## Data Availability

The data that supported the findings are readily available in supplementary material.

## Abbreviations

EDHS: Ethiopian Demographic and Health Survey
FMOH: Federal Ministry of Health of Ethiopia
HC: Health center
km: Kilometer
LARCs: Long-acting reversible contraceptives
DWSIN: Discontinuation while still in need.

## Supplementary Materials

Supplementary Materials Description of supplementary file: the supplementary file comprises of two figures; the first figure is detail about the sampling procedure, and the second is about “Kaplan Meier survival” distribution of the main reasons for Implanon discontinuation.
