# Supplementary material for: Level and Timing of Implanon Discontinuation and Associated Factors among Women Who Used Implanon in Andabet District, Public Health Facilities, North-West Ethiopia
Source: Biomed Res Int. 2021 Aug 6;2021:6647660. doi: 10.1155/2021/6647660 (PMC8363448; doi:10.1155/2021/6647660)
Supplement: Supplementary Materials — Description of supplementary file: the supplementary file comprises of two figures; the first figure is detail about the sampling procedure, and the second is about “Kaplan Meier survival” distribution of the main reasons for Implanon discontinuation. [file 6647660.f1.docx]

**Schematic presentation of sampling procedure (fig1)**

Health institutions in Andabet district

Jaragedo HC (382)

Atsede-Mariam HC (214)

Andabet HC (265)

Genete-Mariam HC (189)

Gono HC (226)

544 women

Note: SRS= systematic random sampling method

*Fig2: Schematic presentation of the sampling frame based on the number of women who requested removal of Implanon for three-months prior to the study, Andabet district, public health facilities, North-West Ethiopia, 2017.*

# Results

**Reasons for Implanon discontinuation**

From the reason of discontinuation women who discontinue secondary to development of side effect were causes for earlier discontinuation compared to discontinue secondary to want to pregnant, and from the side effects women who were develop headache discontinue earliest, followed by pain at insertion arm (fig2).


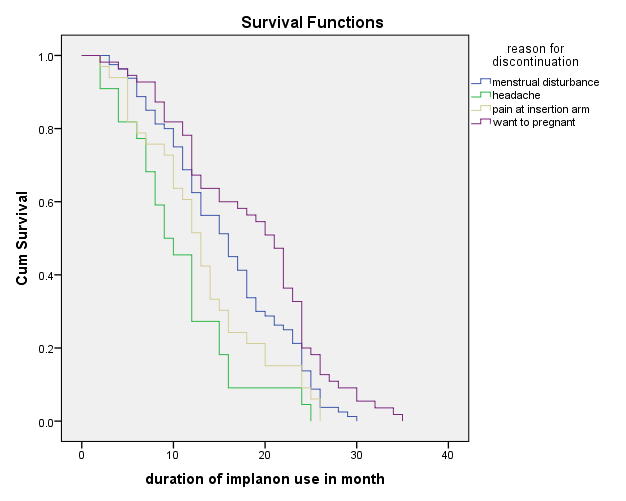


*Fig2: Kaplan Meier survival distribution of the main reasons for implanon discontinuation among women who* requested removal of implanon in Andabet district, public health facilities; North-West Ethiopia: 2017.
